# Subjective executive function deficits in hazardous alcohol drinkers

**DOI:** 10.1177/02698811211032605

**Published:** 2021-07-18

**Authors:** Anna Powell, Harry Sumnall, Cecil Kullu, Lynn Owens, Catharine Montgomery

**Affiliations:** 1 School of Psychology, Liverpool John Moores University, Liverpool, UK; 2 Public Health Institute, Liverpool John Moores University, Liverpool, UK; 3 Liverpool Centre for Alcohol Research, University of Liverpool, Liverpool, UK; 4 Mersey Care NHS Foundation Trust, Liverpool, UK; 5 University of Liverpool, Liverpool, UK

**Keywords:** Cognitive function, executive function, alcohol, binge drinking

## Abstract

**Background:**

Dependent alcohol drinkers exhibit differences in the structure and function of the brain, and impairments in cognitive function, including executive functions (EFs). Less is known about the impact of non-dependent but hazardous use (that which raises the risk of harm), and it is also unclear to what extent executive impairments in this cohort affect real-world function. The current study examines the relationship between alcohol use, EF and alcohol-related problems, in the general population.

**Methods:**

A between-groups cross-sectional design assessed EF across two levels of drinking; hazardous (Alcohol Use Disorders Identification Test (AUDIT) score of ⩾8) and non-hazardous. Alcohol drinkers (*n* = 666; 136 male; 524 female; six not disclosed; aged 28.02 ± 10.40 years) completed validated questionnaires online assessing subjective EF, alcohol use and alcohol-related problems.

**Results:**

Organisation, Strategic Planning, Impulse Control and overall function were significantly impaired in hazardous drinkers. Furthermore, the effect of alcohol on EF, partially mediated the relationship between alcohol use and alcohol-related problems.

**Conclusion:**

Hazardous drinking was associated with lower subjective EF, and this mediated the effect of alcohol on alcohol-related problems. This may be due to changes in prefrontal brain regions, which could indicate greater risk for the development of alcohol dependence (AD). Future research should use additional means to assess EF in hazardous drinkers, including recovery of function, development of AD and the relationship between cognition and alcohol-related daily problems.

## Introduction

Globally, harmful alcohol use is estimated as the seventh leading risk factor for premature death/disability (Griswold et al., 2018). Alcohol-related harm is estimated to cost the NHS £3.5 billion a year (Public Health England, 2014). In the UK in 2018, 7551 deaths were related to alcohol-specific causes (Office for National Statistics, 2019), and in England, there were approximately 358,000 directly alcohol-attributable hospital admissions (NHS Digital, 2020).

Acutely, alcohol acts on GABAergic receptors to potentiate gamma aminobutyric acid (GABA) release, inducing inhibitory sedative effects, and also inhibits glutamatergic receptors, suppressing excitatory glutamate release (Abrahao et al., 2017; Lovinger and Roberto, 2013; Zorumski et al., 2014). Both neurotransmitters contribute to prefrontal cortex (PFC) working memory (WM) processes (Bañuelos and Wołoszynowska-Fraser, 2017). Processes impaired by acute alcohol intoxication include executive functions (EFs; Day et al., 2015), higher-order cognitive functions that govern goal-directed action (Hughes, 2013). Well-supported EF models propose clearly separable, yet related processes (Miyake et al., 2000) with response inhibition (inhibiting dominant behavioural response), task shifting (transferring cognitive resources between tasks) and updating WM (replacing outdated information) emerging as key domains (Diamond, 2013; Miyake and Friedman, 2012). Together, these domains enable critical abilities, such as reasoning, formulating goals, sustained attention, motivation and the flexibility to adapt plans if circumstances change (Aron, 2008). However, although there is generally agreement on these core functions, there is no single accepted definition of EF (Goldstein and Naglieri, 2014), other than that EF is multidimensional (Otero and Barker, 2014), with various processes covered by the ‘umbrella term’ (Chan et al., 2008).

Response inhibition is impaired in acute alcohol use (Day et al., 2015; Field et al., 2010) and associated with decreased brain activity in EF-implicated regions, including the lateral PFC (Anderson et al., 2011). Furthermore, alcohol dependence (AD) is associated with multiple EF impairments linked to prefrontal brain changes (Abernathy et al., 2010; Chanraud et al., 2006; Noel, 2002), which can predict treatment outcomes (Domínguez-Salas et al., 2016). Meta-analysis suggests inhibition in particular is impaired in AD (Smith et al., 2014), and it may be an important factor in developing AD (Holcomb et al., 2019). While EF deficits in AD are well-documented, less is known about the relationship between non-dependent hazardous drinking and EF, how this affects daily life, or how deficits compare to those in AD and could influence drinking behaviour and the development of AD.

The definition of hazardous drinking can vary, but the National Institute for Health Clinical Excellence (2010) defines it as alcohol use that increases risk of harm, which is how it is interpreted in the current study. It is often defined similarly to heavy drinking; both relate to consumption that may increase risk and exceed a specific threshold (Reid et al., 1999). Current UK guidelines recommend ⩽14 units per week, spread evenly over three or more days (Department of Health, 2016). Consequently, drinking patterns that could identify a person as increased risk (Hatton et al., 2009) include drinking over 14 units continuously across the week, or consuming large amounts during drinking sessions (heavy episodic drinking (HED); Wechsler and Nelson, 2006, or ‘binge drinking’; Adan et al., 2017). Such behaviours are included in many alcohol screening tools, including the Alcohol Use Disorders Identification Test (AUDIT; Saunders et al., 1993) used in the current study, with higher scores indicating increased risk.

A systematic review of seven studies investigating EF in heavy drinking reported inconsistent findings, and the meta-analysis found no overall EF impairment (Montgomery et al., 2012). However, their subsequent cross-sectional experimental study of 41 young adults found heavy drinkers (identified using AUDIT data median split) performed worse on all EF tasks: inhibition, shifting, updating and access to semantic memory. Similarly, a more recent systematic review concluded that HED in young adults is associated with poor inhibitory control, and that there is tentative support for deficits in shifting and updating (Carbia et al., 2018b).

In contrast, Carbia et al. (2018a) followed 63 young adults (from age 18) for 11 years and found continuous HED (continuous scores of ⩾4 on AUDIT-Consumption, AUDIT-C) associated with poor inhibition (Stroop Test) and updating (self-ordered pointing test, SOPT), but not shifting (trail making task, TMT). This was not supported in a later cross-sectional study of EF, drinking motives, alcohol use, heavy drinking and related problems (e.g. regretted sexual activity) in 801 21–35-year olds (Martins et al., 2018). They found no association between heavy drinking and inhibition or updating, and no EF components predicted alcohol-related problems. Interestingly, better shifting-specific abilities associated with heavy drinking. While this appears counterintuitive, strong shifting-specific abilities differ from other EF by undermining self-control (Friedman and Miyake, 2017; Herd et al., 2014). Known as the ‘stability-flexibility trade-off’, high shifting enables moving attention to appealing alternatives, but impairs maintenance/shielding of long-term goals (Hofmann et al., 2012).

Others have found impaired response inhibition in HED young adults on Go/NoGo task (Ames et al., 2014; Czapla et al., 2015; Lannoy et al., 2020). Furthermore, Lannoy et al. (2019b) and Kim and Kim (2019) also found that in young HED adults, inhibition performance on the Flanker task was impaired compared to controls, though shifting (Number Letter task) and updating (Letter Memory task) abilities were not. The authors suggested that this highlighted the importance of inhibitory control in alcohol use, and that a distinction between binge and dependent drinking may be lack of a ‘general’ executive deficit.

However, many researchers have also found hazardous drinkers do not differ significantly to controls on EF task performance. This includes on Go/NoGo tasks assessing inhibition (Blanco-Ramos et al., 2019; Lannoy et al., 2017; López-Caneda et al., 2012, 2014), and *n*-back tasks, which assess updating (Park and Kim, 2018; Schroder et al., 2019). A possible explanation for these discrepancies is a ‘neurocompensatory mechanism’ in young drinkers, in which increased cognitive effort enables performance preservation, which loses efficiency over time and continued hazardous drinking (Almeida-Antunes et al., 2021; Gil-Hernandez et al., 2017; Tapert et al., 2004). Indeed, the Go/NoGo studies above all found electrophysiological differences in hazardous drinkers, including delayed latencies and/or higher amplitudes of event-related potentials (ERPs) indexing executive control. Furthermore, Smith and Mattick (2013) found hazardous drinkers had poorer Stop Signal Task inhibition, but higher P3 amplitudes on successful versus failed trials. A critical review by Lannoy et al. (2019a) noted studies showing reduced electrophysiological activities indexing attentional/executive processes (e.g. Maurage et al., 2009, 2012) are typically those using less executive experimental paradigms. Additionally, functional neuroimaging reveals that while decreased activity in frontoparietal areas during EF tasks may be a precursor for hazardous drinking, these areas often display hyperactivation during EF tasks *after* the onset of this (Lees et al., 2019; Spear, 2018).

Structural neuroimaging indicates that HED (determined by questions on consumption speed and frequency of 6+ drinks in one occasion, or Alcohol Use Questionnaire (Mehrabian and Russell, 1978) questions on HED frequency) is associated with whole-brain white matter degradations, and anomalies in prefrontal grey matter (Doallo et al., 2014; Smith et al., 2017). This was linked to poor updating on the Cambridge Neuropsychological Test Automated Battery (CANTAB) Spatial Working Memory test and the non-computerised version, the SOPT. However Smith et al. (2017) found no relationship between white matter degradation and the inhibition assessed by the CANTAB Stop Signal Task.

While EF has been investigated in hazardous drinkers using behavioural paradigms and neuroimaging, few studies have addressed the effects of alcohol on EF by using subjective assessments. This becomes interesting especially when one considers that increased cognitive effort to achieve satisfactory performance (as in the neurocompensation hypothesis) may be better reflected in self-report assessment of difficulties. Research using subjective measures is conflicting, with Heffernan et al. (2004) finding that excessive drinkers experienced more problems related to the executive component of memory. Similarly, Houston et al. (2014) found greater alcohol use associated with poorer EF measured by subjective EF (Dysexecutive Functioning Questionnaire), and task performance (TMT, Go/NoGo and Wisconsin Card Sorting Test). However, Czapla et al. (2015) found that HED and controls did not differ in overall response inhibition on a Go/NoGo task, or self-reported impulsiveness, though there was an impairment on the task for alcohol-related stimuli.

Finally, hazardous drinking has a considerable effect on overall function and quality of life, including on interpersonal relationships, finances and employment (World Health Organization, 2004). The relationship between alcohol use and EF may contribute to this, as EF affects much of everyday life (Snyder et al., 2015), and EF dysfunction in AD decreases quality of life (Brion et al., 2017). However, there is little evidence of how this relates to non-dependent hazardous drinking. One study of 62 college students found EF mediated the relationship between alcohol use and overall life functioning (assessed by the Barkley Functional Impairment Scale); however, this was in an ADHD population predisposed to EF deficits (Langberg et al., 2015). Another study found a small dose effect with the heaviest drinkers (10+ drinks a week) demonstrating lower general cognitive function and poor reported daily life functioning (Hendrie et al., 1996). While this supports a relationship between daily functioning and the effect of hazardous drinking on cognitive function, it did not specifically examine EF. In contrast, Martins et al. (2018) found no relationship between EF and alcohol-related problems.

Clearly, EF is affected by hazardous drinking to some extent, but the aetiology is not always consistent. This could be due to neurocompensation in individuals, which may be better reflected in subjective judgement of EF. Furthermore, while EFs are predictive of clinical outcomes in AD, less is known about the relationship between EF and daily-life outcomes in the general population. The current study investigated subjective EF deficits in adult non-dependent hazardous drinkers using an online survey and explored the relationship between deficits and self-reported alcohol-related problems. Based on the literature above, we hypothesised that (1) hazardous drinkers would have significantly poorer subjective EF than non-hazardous drinkers, and (2) the relationship between alcohol use and alcohol-related problems would be mediated by the effect of alcohol on subjective EF.

## Methods

### Design

A factorial design assessed EF between male and female hazardous and non-hazardous drinkers. The independent variables were alcohol use with two levels; non-hazardous and hazardous drinking (determined by AUDIT cut-off score; ⩾8 deemed hazardous drinking; World Health Organization, 2001), and gender with two levels – male and female. The main dependent variable was EF.

### Participants

Eight hundred and three individuals took part. Upon initial screening, 128 incomplete datasets were removed (15.9%), and nine more were removed as outliers.^1^ Thus, the study comprised of 666 participants (136 male; 524 female; six gender not disclosed; aged 28.02 ± 10.40 years). Participants were recruited globally (73.6% UK, 9.6% Ireland, 6.2% USA, 2.6% Australia and 7.7% rest of world). Participants were categorised into non-hazardous (*n* = 323 (48.50%); 56 male, 264 female; three gender not disclosed, aged 29.73 ± 10.68 years; mean AUDIT total score = 4.72, SD = 1.77) and hazardous (*n* = 343 (51.50%); 80 male, 260 female; three gender not disclosed, aged 26.40 ± 9.85 years; mean AUDIT total score = 13.04, SD = 4.80) drinkers, using AUDIT score (⩾8 deemed hazardous).

Recruitment channels included an advert on the Liverpool John Moores University (LJMU) website and personal/professional social media, referrals from previous participants, research team acquaintances and an email to LJMU students. Each advert contained a link to the Qualtrics survey. Potential participants self-identified as eligible if they were alcohol drinkers aged 18+. There were no exclusion criteria. The original recruitment target was 282 participants, based on a multivariate analysis of variance (MANOVA) sample size calculation with a 95% confidence level (*f*^2^ ⩾ 0.02, a small effect size; Cohen, 2013) using GPower version 3.1.94 (Heinrich Heine – Universitat Dusseldorf, Germany) (Faul et al., 2009), adjusted for MANCOVA (Dattalo, 2008).

### Materials

#### Demographics

Participants answered questions on age, gender, country of residence, employment status, education level, housing status, mental health diagnoses and medication.

#### Executive function

This study used the Executive Function Index (EFI; Spinella, 2005) which is a 27-item, five-point Likert-scale questionnaire assessing five EF components derived from factor analysis; Motivational Drive, Strategic Planning, Organisation, Impulse Control and Empathy. Motivational Drive items assess interest in novelty, activity level and behavioural drive. Strategic Planning items measure ability to use strategies, plan and think ahead. Organisation assesses sequencing, multitasking and holding information in the WM to inform decisions. Impulse Control measures self-inhibition, social conduct and risk taking. Empathy items assess prosocial behaviours, a cooperative attitude and concern for others’ wellbeing.

Higher total (global measure) and subscale scores indicate better EF. Subscale scores are calculated by summing relevant items (taking account of reverse scoring). The EFI corresponds well with neuroanatomical findings (Spinella, 2005), and also into a three-factor model, in which Impulse Control and Empathy form one factor, Strategic Planning and Organisation another and Motivational Drive a third. These correspond to the model of functional organisation of orbitofrontal, dorsolateral and medial prefrontal circuits (Cummings, 1993; Miller and Cummings, 2017). In initial development, EFI had a Cronbach’s α ranging between 0.69 and 0.76 for the five subscales, with a total α of 0.82, an acceptable internal consistency (Spinella, 2005). In our study, Cronbach’s α ranged from 0.76 to 0.80 across the items, and a total α of 0.76. It was lower for the subscales, ranging from 0.55 to 0.63, and a total of 0.63.

#### Mood state

The Hospital Anxiety and Depression Scale (HADS) was used to assess state Anxiety and Depression (Zigmond and Snaith, 1983). HADS is a four-point, 14-item Likert-scale, scored 0–3 by separately summing subscales (some items require reverse scoring). Condition boundary points for both subscales are; 8–10 = mild, 11–14 = moderate and 15–21 = severe. A general population review of 747 studies found HADS demonstrates good validity and reliability (Bjelland et al., 2002).

#### Alcohol use

The AUDIT is a 10-item five-point Likert-scale assessing harmful/hazardous drinking developed by the World Health Organization (Saunders et al., 1993). A cut-off score of 8+ is recommended as an indicator of hazardous/harmful alcohol use, and possible alcohol dependence (World Health Organization, 2001), and so in this study, participants were grouped as scoring <8 (non-hazardous) or ⩾8 (hazardous). In addition, a composite score of the first three questions can be used to assess level of alcohol consumption, classed as the AUDIT-C scale (Bradley et al., 2007). The AUDIT is reliable (Donovan et al., 2006; Fiellin et al., 2000) and validated within primary health care in six countries (World Health Organization, 2001) and the general population (Aalto et al., 2009). Indeed, a systematic review by Fiellin et al. (2000) concluded that the well-used cut-off of 8 for the AUDIT is more sensitive for identifying hazardous and harmful drinkers than two other measures – CAGE (Ewing, 1984) and Short Michigan Alcoholism Screening Test (Selzer et al., 1975).

#### Alcohol-related problems

The Alcohol Problems Questionnaire (APQ) by Drummond (1990) is a 44-item tool rated yes(1)/no(0), contributing to a common score, and eight separately summed subscales. Five subscales apply to all participants: the perceived drinking impact on Financial, Legal, Physical, Social and Psychological issues. The Alcohol Problems Questionnaire Common (APQC) score is comprised of total scores of these five subscales and demonstrates high reliability coefficients, internal consistency and stability over time (Drummond, 1991; Williams and Drummond, 1994). Where relevant, subscales of impact on Work, relationships with Children and Spouse are also assessed. Lower scores within each subscale indicate fewer alcohol-related problems. APQ demonstrates high test–retest reliability (Williams and Drummond, 1994) that has been validated within a clinical population (Drummond, 1990; Williams and Drummond, 1994) and a sample of college students (Drummond, 1991) and is the UK measure of choice for alcohol-related problems (Raistrick et al., 2019).

### Procedure

Potential participants read the online study information and confirmed eligibility. They were reminded of confidentiality, right to withdraw, or omit questions, and provided consent through a tick-box. When finished, participants were provided with a full debrief, with no reward for completion, but could enter a prize draw for one of three shopping vouchers. This study was approved by LJMU Research Ethics Committee.

### Statistical analyses

All analyses were completed using SPSS v26 (IBM Corp., Armonk, NY, USA). Factorial MANOVA assessed mood state (HADS Anxiety and Depression scores) across gender and drinking level. A 2×2 Factorial MANCOVA was then performed on EFI subscales (dependent variables assessing EF), with drinking category (non-hazardous and hazardous) and gender (male and female) as the between-groups independent variables. Mood state and age were included in the model as continuous covariates, chosen due to their associations with EF (Best and Miller, 2010; Grissom and Reyes, 2019; Gulpers et al., 2016; Snyder, 2013; Zaninotto et al., 2018) and alcohol use (Jane-Llopis and Matytsina, 2006; Mooney et al., 1987; Wilsnack et al., 2009).

Finally, a hierarchical multiple regression was conducted with alcohol use (AUDIT-C) and EF (EFI subscales) as predictors of alcohol-related problems, with a subsequent mediation analysis, using the PROCESS plugin version 3.5, as in Hayes (2017), examining the mediation of EF (EFI total score) on the relationship between alcohol use (AUDIT-C) and related problems (APQC). Mood state, age and gender were included in the mediation as covariates, which was further supported by their significant contributions in the Factorial MANCOVA.

## Results

Table 1 shows descriptives for mood state and alcohol problems.

**Table 1.** Adjusted means for anxiety and depression, and unadjusted APQ means, by gender and drinking level.

Hospital anxiety and depression scale (MANOVA)	Anxiety		Depression													
	M	SE	M	SE												
Drinking level																
Non-hazardous	7.94	0.31	3.87	0.24												
Hazardous	8.80	0.27	4.24	0.21												
Gender																
Male	7.46*	0.37	3.92	0.28												
Female	9.28	0.19	4.19	0.14												
Alcohol problems questionnaire (unadjusted)	Friendships		Partner		Children		Work		Money		Legal		Physical		Psychological	
	M	SD	M	SD	M	SD	M	SD	M	SD	M	SD	M	SD	M	SD
Drinking level																
Non-hazardous	0.09	0.29	0.11	0.41	0.02	0.13	0.16	0.37	0.20	0.40	0	0	0.84	1.07	0.29	0.78
Hazardous	0.71	0.80	1.12	1.63	0.24	0.78	0.44	0.86	0.36	0.79	0.03	0.17	1.74	1.38	0.41	0.78
Gender																
Male	0.50	0.66	0.67	1.01	0.04	0.20	0.38	0.88	0.25	0.53	0.04	0.20	1.17	1.31	0.42	0.83
Female	0.26	0.59	0.42	1.20	0.12	0.57	0.32	0.65	0.26	0.56	0	0	1.18	1.26	0.30	0.76

MANOVA: multivariate analysis of variance.

Mood state = hospital anxiety and depression scale anxiety and depression scores; alcohol problems = alcohol problems questionnaire scores; hazardous drinking = alcohol use disorders identification score of ⩾8.

* *p* < 0.0001.

Factorial MANOVA assessed differences in state anxiety and depression (HADS) across gender and drinking level (see Table 1).^2^ The Levene’s and Box’s tests were acceptable (*p* < 0.05). There was a significant main effect of gender [*F*(2, 651) = 11.50, *p* < 0.0001, Wilks’ Λ = 0.966, η_p_^2^ = 0.03], but not drinking level [*F*(2, 651) = 2.14, *p* = 0.12, Wilks’ Λ = 0.993, η_p_^2^ = 0.01], and no significant interaction between the two factors [*F*(2, 651) = 0.07, *p* = 0.94, Wilks’ Λ = 1.00, η_p_^2^ = 0.00]. Pairwise comparisons revealed that females had significantly higher state anxiety than males [*F*(1, 652) = 19.47, *p* < 0.0001, η_p_^2^ = 0.03], but that there was no gender difference for state depression (*p* = 0.39).

### Executive function

For the factorial MANCOVA, scatterplots indicated approximately linear relationships between each pair of dependent variables, and between the covariates and each dependent variable. Homogeneity of regression was achieved at *p* > 0.05 for covariate by drinking level interaction, covariate by gender interaction and covariate by drinking level by gender interaction, in all cases. The Levene’s test indicated the homogeneity of variance assumption was met for all EFI subscales between groups (*p* > 0.05). The Shapiro–Wilk tests with a Bonferroni correction indicated residual normality was met for 18 out of 20 conditions (*p* > 0.003), which was deemed acceptable. The Box’s test of equality of covariance matrices was met (*p* = 0.12).

The 2×2 factorial MANCOVA (see Table 2) found a significant effect of each covariate on EFI scores: age (*F*(5, 615) = 11.34, *p* < 0.0001, Wilks’ Λ = 0.916, η_p_^2^ = 0.08), depression (*F*(5, 615) = 38.97, *p* < 0.0001, Wilks’ Λ = 0.759, η_p_^2^ = 0.24) and anxiety (*F*(5, 615) = 11.70, *p* < 0.0001, Wilks’ Λ = 0.913, η_p_^2^ = 009). After controlling for these, there was a significant difference between drinking level groups on EFI scores (*F*(5, 615) = 12.90, *p* < 0.0001, Wilks’ Λ = 0.905, η_p_^2^ = 010). Gender was also included in the model as a fixed factor, displaying a significant effect on EFI scores (*F*(5, 615) = 4.50, *p* = 0.0002, Wilks’ Λ = 0.961, η_p_^2^ = 0.04); however, there was no significant interaction between gender and drinking level (*F*(5, 615) = 0.34, Wilks’ Λ = 0.997, *p* = 0.89, η_p_^2^ = 0.00).

**Table 2.** Adjusted means for executive function index (EFI) subscales, by drinking level and gender, controlling for mood state and age.

	Motivational drive		Organisation		Strategic planning		Impulse control		Empathy	
	M	SE	M	SE	M	SE	M	SE	M	SE
Drinking level										
Non-hazardous	14.05	0.17	16.87*	0.23	25.54***	0.24	16.43***	0.21	26.01	0.18
Hazardous	14.03	0.15	16.15	0.20	23.90	0.21	14.62	0.19	26.11	0.16
Gender										
Male	13.86	0.21	16.48	0.28	24.44	0.28	14.94***	0.25	25.68**	0.22
Female	14.22	0.10	16.54	0.14	24.99	0.14	16.11	0.13	26.43	0.11

Subjective executive function = executive function index subscales (motivational drive, organisation, strategic planning, impulse control, empathy); hazardous drinking = alcohol use disorders identification score of ⩾8; mood state = hospital anxiety and depression scale anxiety and depression scores.

From smallest, *p < 0.05. **p < 0.01. ***p < 0.001.

Hazardous drinkers had lower scores on all EFI subscales (with the exception of Empathy); differences were significant for EFI subscales Organisation (*F*(1, 619) = 5.44, *p* = 0.02, η_p_^2^ = 0.01), Strategic Planning (*F*(1, 619) = 27.53, *p* < 0.0001, η_p_^2^ = 0.04) and Impulse Control (*F*(1, 619) = 41.91, *p* < 0.0001, η_p_^2^ = 0.06]) There was no significant difference between drinking level groups on the Motivational Drive and Empathy subscales (*p* = 0.93 and 0.70, respectively). Therefore, hazardous drinking was associated with worse subjective EF compared to non-hazardous drinking.

Males had lower scores on all EFI subscales, but this difference was significant for EFI subscales Impulse Control (*F*(1, 619) = 16.77, *p* < 0.0001, η_p_^2^ = 0.03], and Empathy (*F*(1, 619) = 9.57, *p* = 0.002, η_p_^2^ = 0.02). There were no differences between males and females on the Motivational Drive, Organisation and Strategic Planning subscales (*p* = 0.12, 0.86 and 0.09, respectively). Therefore, males had worse subjective EF compared to females.

## Relationship between subjective executive function and real-life alcohol-related problems

A hierarchical regression modelled the relationship between EF and alcohol-related problems, with continuous APQC score as the dependent variable. Variables were entered simultaneously in successive model blocks: demographic variables (age and gender) in model one, alcohol use (AUDIT-C scores and expected to account for the most variance) in model two, mood state (HADS Depression and Anxiety scores) in model three and EFI subscales (Motivational Drive, Impulse Control, Organisation, Strategic Planning and Empathy) in model four, thereby ensuring that cognitive factors were added successively. Model parameters are shown in Table 3.

**Table 3.** Hierarchical multiple regression parameters with alcohol problems questionnaire common score as the dependent variable.

	Unstandardized and standardized coefficients			Squared semi-partial correlation coefficients	Obtained I and I values		Obtained I values			
	B	SE B	β	*sr* ^2^	*t* ^a^	*P*	*R*	*R* ^2^	*∆R* ^2^	*p*
Model 1							0.226	0.051	0.051	<0.0001
Constant	6.231	0.714			8.725	<0.0001				
Age	−0.069	0.012	−0.231	0.051	−5.722	<0.0001				
Gender	−0.345	0.307	−0.045	0.002	−1.122	0.262				
Model 2							0.468	0.219	0.168	<0.0001
Constant	1.871	0.752			2.488	0.013				
Age	−0.054	0.011	−0.183	0.031	−4.948	<0.0001				
Gender	0.172	0.283	0.023	0.000484	0.609	0.543				
AUDIT-C	0.599	0.052	0.417	0.168	11.437	<0.0001				
Model 3							0.626	0.392	0.173	<0.0001
Constant	−0.048	0.688			−0.070	0.944				
Age	−0.046	0.010	−0.156	0.022	−4.716	<0.0001				
Gender	−0.029	0.252	−0.004	0.000016	−0.115	0.908				
AUDIT-C	0.568	0.046	0.395	0.151	12.246	<0.0001				
Anxiety	0.111	0.028	0.155	0.016	3.964	<0.0001				
Depression	0.298	0.036	0.313	0.068	8.207	<0.0001				
Model 4							0.666	0.444	0.052	<0.0001
Constant	3.985	1.427			2.792	0.005				
Age	−0.025	0.010	−0.084	0.006	−2.520	0.012				
Gender	0.138	0.246	0.018	0.000289	0.562	0.574				
AUDIT-C	0.470	0.048	0.327	0.088	9.743	<0.0001				
Anxiety	0.072	0.028	0.100	0.006	2.558	0.011				
Depression	0.217	0.040	0.223	0.026	5.307	<0.0001				
Motivational drive	−0.061	0.043	−0.052	0.002	−1.415	0.158				
Organisation	−0.108	0.033	−0.122	0.010	−3.277	0.001				
Strategic planning	−0.011	0.033	−0.012	0.0001	−0.329	0.742				
Impulse control	−0.190	0.036	−0.196	0.025	−5.223	<0.0001				
Empathy	0.083	0.041	0.067	0.004	2.003	0.046				

Depression and anxiety = hospital anxiety and depression scale subscales; motivational drive, organisation, strategic planning, impulse control and empathy = Executive Function Index subscales.

AUDIT-C: Alcohol Use Disorders Identification Test-Consumption.

a Model 1: *df* = 608; model 2: *df* = 607; model 3: *df* = 605 and model 4: *df* = 600.

Model one significantly predicted alcohol-related problems *F*(2, 608) = 16.38, *p* < 0.0001, as did model two *F*(3, 607) = 56.85, *p* < 0.0001 and model three *F*(5, 605) = 78.13, *p* < 0.0001. For these three models, gender was not a significant predictor. Finally, model four also significantly predicted alcohol-related problems *F*(10, 600) = 47.92, *p* < 0.0001 (though gender, Motivational Drive, Strategic Planning, and Empathy were not significant predictors). The addition of EFI subscales explained an additional 44% of the variance, taking overall explained variance in alcohol-related problems to 44.4%. Beta coefficients and partial correlations indicated that in model four, predictor order of importance was as follows: alcohol use, state depression, Impulse Control, Organisation, state anxiety and age (β = 0.327, 0.223, −0.196, −0.122, 0.100 and −0.084, respectively, *p*-values <0.05). The final model effect size was calculated as *f*^2^ = 0.80, a large effect (Cohen, 1988), and the local effect size of the EFI subscales was calculated at *f*^2^ = 0.094 (using local effect size calculation proposed by Selya et al., 2012), a small effect.

**Figure 1.**
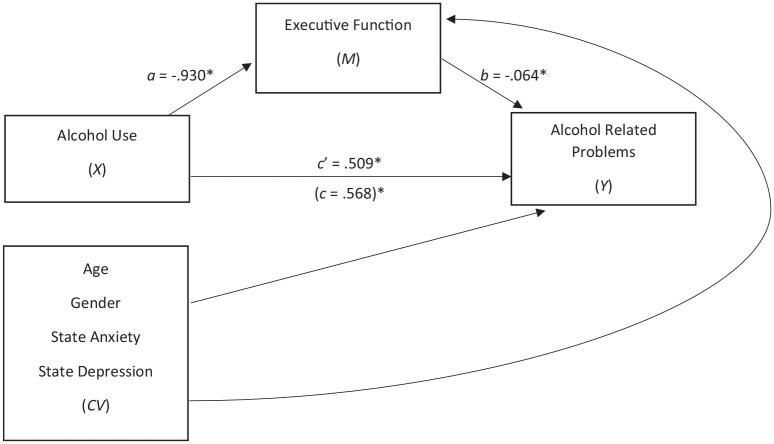
The mediating effect of executive function (EF) on the relationship between alcohol use and alcohol related problems, while controlling for age, gender, and state anxiety and depression. Note: All presented effects are unstandardized; *a* is effect of alcohol use (alcohol use disorders identification test-consumption) on EF; *b* is effect of EF (executive function index total score) on alcohol-related problems (alcohol problems questionnaire common); *c’* is direct effect of alcohol use on alcohol-related problems; *c* is total effect of alcohol use on alcohol-related problems. State anxiety and depression = hospital anxiety and depression scale subscales. *p < 0.001.

Mediation analysis was then used to assess the relationship between alcohol use, EF and alcohol problems. This indicated that alcohol use (AUDIT-C) was indirectly related to alcohol-related problems (APQC) through its relationship with EF (EFI total score), after controlling for covariates. As shown in Figure 1, EF mediated the relationship between alcohol use and alcohol-related problems. Higher consumption was associated with poorer EF (*a* = 0.930, *p* < 0.001; standardized *a* = −0.205), which was subsequently related to more alcohol-related problems (*b* = −0.064, *p* = 0.001; standardized *b* = 0.203). A 95% bias-corrected confidence interval based on 10,000 bootstrap samples indicated the indirect effect, *ab* = 0.060, BCa CI [0.033, 0.091] was statistically significant. However, the direct effect of alcohol use on alcohol-related problems was also significant *c’* = 0.509, *p* < 0.001, indicating partial mediation of EF. The completely standardized indirect effect was ab_cs_ = 0.042, BCa CI [0.024, 0.063].

## Discussion

The current study examined drinking behaviour and EF. Hypothesis one was partially supported as some EFI subscales (Strategic Planning, Impulse Control, and Organisation) were significantly lower in hazardous drinkers, indicating poorer performance. Hypothesis two was also supported, as EF partially mediated the relationship between alcohol use and alcohol-related problems.

After controlling for covariates, hazardous drinking was associated with worse EFI Strategic Planning, Impulse Control, and Organisation, but not Empathy and Motivational Drive. This suggests hazardous drinkers in this study struggle with planning/using strategies, self-inhibition, risk taking and holding information in mind or multitasking, but not prosocial behaviours or motivation. This supports research showing EF deficits in hazardous drinkers (Doallo et al., 2014; Martins et al., 2018; Smith et al., 2017), particularly in inhibition (Ames et al., 2014; Carbia et al., 2018a, 2018b; Czapla et al., 2015; Kim and Kim, 2019; Lannoy et al., 2019b, 2020; Montgomery et al., 2012), as Impulse Control was the largest subscale deficit found.

This highlights potential similarities between EF in hazardous drinking, and AD such as in Smith et al. (2014). Furthermore, these results may contrast with those showing no inhibitory deficit in hazardous drinking (Blanco-Ramos et al., 2019; Czapla et al., 2015; Lannoy et al., 2017; López-Caneda et al., 2012, 2014; Martins et al., 2018; Smith et al., 2017) due to the varied age range; 48.4% of participants were above 24 years old, which has been proposed as a more appropriate ‘end of adolescence’ in relation to various biological and social factors, including neurodevelopment (Sawyer et al., 2018). It is therefore possible to infer that the current sample was diverse with regard to neurological development (and years of continuous hazardous drinking), which may have reduced the ability of neurocompensation to preserve inhibition, contrasting with studies focusing on young adults. These results also support a possible distinction from AD as reported in Kim and Kim (2019), as not every EFI subscale was significantly poorer in hazardous drinkers. Importantly, poor EF (particularly inhibition) appears to be involved in the development and maintenance of addictions, including AD (Hester et al., 2010). Results such as the current study therefore indicate a potentially vulnerable cohort. However, it is likely the relationship between EF and alcohol use is cyclical, with elements of EF being heritable and increasing risk of problematic drinking (Benzerouk et al., 2013).

The current findings may result from anomalies in prefrontal structures; indeed, the EFI subscales differentially associate with three prefrontal EF systems (Cummings, 1993; Miller and Cummings, 2017); Impulse Control and Empathy with orbitofrontal, Strategic Planning and Organisation with dorsolateral, and Motivational Drive with medial (Miley and Spinella, 2006). These areas are disrupted in AD, associated with decreased EF (Abernathy et al., 2010). This is partially reversible with long-term abstinence, but to what extent is unclear (Moselhy et al., 2001). Less is known about hazardous drinking and neural function, though as discussed, there is evidence HED leads to prefrontal anomalies associated with impaired EF (Doallo et al., 2014; Smith et al., 2017).

Specific subscale impairments indicate more potential damage to orbitofrontal and dorsolateral regions, which may differentiate hazardous and dependent drinkers. There is evidence to suggest hazardous drinking cessation leads to partial cognitive and neural recovery, though not to the same level as control participants (Lees et al., 2019). However, such interpretation of the results with regard to brain structure/function is speculative, due to the nature of the assessments used. Future EF research should use additional paradigms (neuroimaging, ERP and objective EF assessments) to investigate changes in the brain structure/function of hazardous drinkers, the cause/effect, reversibility or chronic nature of any changes and predictability of assessments to indicate risk of progression from hazardous drinking to AD.

Our second prediction was supported as hazardous drinking predicted alcohol-related problems, and this was partially mediated by EF. Although the APQC score does not indicate specific issues, its high internal consistency indicates problems assessed within it may co-occur, indicating general problematic tendencies (Drummond, 1991). It is understandable how problems planning/using strategies, self-inhibiting, managing risk taking and holding information in mind or multitasking could contribute to items included in APQC. Indeed, hazardous drinkers (⩾8 AUDIT score) experience more mental health problems, hospital admissions and social issues (Conigrave et al., 1995), and alcohol use contributes to financial, legal and workplace problems (Rehm, 2011). EF is associated with all of these domains (Allan et al., 2016; Gulpers et al., 2016; Spinella et al., 2004; Snyder, 2013; Wolf, 2010; Yeh, 2013), so it is possible alcohol-related EF impairments may partially underlie the disruptive impact of problematic drinking for some people, even before considering whether hazardous drinking/poor EF increases risk of AD. Further research could examine which alcohol-related problems are mediated by EF (and by which EF specifically) and consider whether this knowledge could be used to reduce alcohol-related problems (e.g. through EF training or other interventions).

This study had a number of limitations. Conducted during the first 2020 COVID-19 lockdown, this may have induced drinking pattern changes due to stress/boredom (Institute of Alcohol Studies, 2020). Indeed, a general population survey suggested 21% of UK adults reported drinking more than normal, whereas 35% reduced/abstained (Alcohol Change UK, 2020). Another large self-selecting online survey (*n* = 40,000) found 44% of respondents reported an increase in drinking (Global Drugs Survey, 2020), and 23.8% reported an increase in HED (though 30.5% of these said this increase was slight). However, the Alcohol Change survey found people whose drinking increased were those who already drank heavily prior to the lockdown. Furthermore, during lockdown, drinking may be somewhat different, the AUDIT asks questions in relation to the previous 12 months, so classification of drinking group should have remained stable.

We also aimed to keep the survey short to increase engagement; thus, no data were collected on abstinence period from alcohol. It is possible participants experienced alcohol acute/sub-acute effects (such as residual intoxication), which may have impacted their responses. However, as hazardous drinkers had higher overall alcohol consumption and were the group demonstrating poorer EF, the effects found are unlikely related to sub-acute intoxication, even if this occurred for some people. Statistical limitations include the lower Cronbach’s α coefficients for subscales of the EFI, indicating potential internal inconsistencies and future research should seek to use additional methods of EF assessment. Additionally, as this was a cross-sectional survey, it was not possible to discern whether lower EF was a cause or effect of hazardous drinking in this cohort.

Finally, the lockdown and survey-length restrictions also influenced the type of data that could be collected; hence, the study only included self-report measures and not objective assessments as a measure of comparison. While all measures used are well-validated, it is possible that self-report assessment of EF may be more vulnerable to inaccuracies as a result of alcohol effects on metacognition (Le Berre et al., 2017), or due to other uncontrolled extraneous factors, such as education (Spinella and Miley, 2003) or personality (Buchanan, 2016). We also had no control over time of testing. As EF displays diurnal variations and individual differences resulting from circadian typology (Adan, 1993), future studies should control for time of testing and include the use of objective EF measures, such as validated experimental tasks.

Despite these limitations, this study highlights the nature of EF deficits in hazardous drinking, and the mediating effect of EF and drinking on real-world functioning, suggesting hazardous drinkers may be more vulnerable. Research has shown EFs can be improved via intervention (Diamond and Ling, 2016). Furthermore, EF training has successfully reduced alcohol consumption in hazardous drinkers (Houben et al., 2011a, 2011b, 2012), so a targeted intervention improving EF in a hazardous drinking cohort could reduce the risk of developing AD and other alcohol-related problems.

## Conclusion

In conclusion, the current study examined hazardous drinking and EF. Hazardous drinkers reported significantly lower subjective EF, and the relationship between alcohol use and alcohol-related problems was partially mediated by the effect of alcohol use on subjective EF, indicating the importance of understanding and addressing poorer EF in hazardous drinkers. Further research should use additional methods to assess EF in hazardous drinking, including recovery of function, study whether this contributes to AD development (and if this is predictive), examine which alcohol-related problems are mediated by EF, and to consider options for interventions.

## Notes

1. Inspection of Mahalanobis Distance and Standardized Residuals during the main analysis identified nine outliers (three male; six female; three non-hazardous; six hazardous). These participants were removed from all final analyses and descriptives.

2. The Shapiro–Wilk tests using a Bonferroni correction indicated normality of mood state across gender and drinking level was violated for six out of eight tests (*p* < 0.006). While this suggests the results should be interpreted with caution, due to there being no non-parametric MANOVA equivalent, and due to MANOVA being fairly robust with regard to normality violations, it was decided to continue with this analysis.
